# Enhanced Thermal Performance of Composite Phase Change Materials Based on Hybrid Graphene Aerogels for Thermal Energy Storage

**DOI:** 10.3390/ma15155380

**Published:** 2022-08-04

**Authors:** Yu Shang, Dong Zhang, Minrong An, Zhao Li

**Affiliations:** 1 School of Materials Science and Engineering, Xi’an Shiyou University, Xi’an 710065, China; 2 Key Laboratory of Advanced Civil Engineering Materials, Ministry of Education, School of Materials Science and Engineering, Tongji University, Shanghai 201804, China

**Keywords:** graphene aerogels, phase change materials, thermal conductivity, latent heat

## Abstract

Thermal conductivity and latent heat are crucial performance parameters for phase change materials (PCMs) in thermal energy storage. To enhance the thermal performance of PCMs, with the help of graphene oxide (GO) acting as a dispersing agent, well-defined hybrid graphene aerogels (HGAs) with a three-dimensional (3D) porous structure were successfully synthesized by hydrothermal reaction of GO and graphene nanoplatelets (GNPs). GNPs, dispersing uniformly along the interconnecting graphene network, acted as thermal conductive fillers and supporting materials. Palmitic acid (PA) was impregnated into the HGA by vacuum forces. It was found that the thermal conductivity of the PA/HGA was enhanced without compromising heat storage capacity. Compared with PA, the PA/HGA with 4.2 wt% GNPs exhibited enhanced thermal conductivity of 2.1 W/mK and high latent heat of 206.2 J/g simultaneously. The PA/HGA with good thermal performance has potential applications in thermal energy storage.

## 1. Introduction

Energy shortage is a serious global problem facing the world. Improving the efficiency of energy utilization is a way to alleviate the energy crisis. Phase change materials, with their exploitable ability to store and release large amounts of latent heat, are key materials for thermal energy storage [1]. Organic PCMs, such as paraffin and palmitic acid (PA), have seen substantial use in applications for buildings and thermal management for electronics and solar energy conversion because of their high latent heat, low supercooling degree, and excellent chemical stability [2,3]. The main problems with organic PCMs, however, are their low thermal conductivity and their leakage of PCMs above their melting temperature [4,5].

To overcome these problems, shape-stable PCMs have been prepared using supporting materials with high thermal conductivity, which can improve heat transfer efficiency and keep the shape of PCMs above melting temperature [6,7,8]. This has become one of the most effective ways to improve thermal performance. In past decades, porous materials such as expanded graphite and expanded perlite have been used to prepare shape-stable PCMs [9,10,11,12,13]. The porous structure is conducive to improving the thermal conductivity of PCMs. However, some issues pertaining to shape-stable PCMs persist and require further study. Most studies to date have focused on improving thermal conductivity rather than on overall performance [14,15,16]. Generally, a high content of supporting materials decreases the latent heat of shape-stable PCMs because the supporting materials do not contribute to latent heat storage. In addition, the enhancement effect in the thermal conductivity of a traditional porous structure has been unsatisfactory. Therefore, excellent supporting materials haves been a key factor to improving the properties of PCMs.

Recently, graphene aerogels (GAs) with ultrahigh thermal conductivity and a three-dimensional (3D) porous structure have been investigated as thermal fillers and supporting materials in PCMs [17,18,19,20,21,22,23,24]. Yang et al. [17] prepared high-quality GAs by freeze-drying the graphene oxide (GO) and high-temperature annealing of the GA at 2800 °C. The composite PCMs with 5 wt% graphene had a thermal conductivity of 4.28 W/mK, 180% higher than that of pure 1-octadecanol. The heat storage capacity decreased slightly to 225.3 J/g. Liu et al. [25] prepared GA/PCMs using the one-pot method. The thermal conductivity of the composite was 3.21 W/mK with 10 wt% reduced GO (RGO). Thus, composite PCMs with enhanced thermal properties can be prepared with GA. Cao et al. [26] studied the influence of GA on the crystallization and phase-change process of paraffin. The contradiction between thermal conductivity and heat storage capacity could be adjusted by controlling the porosity and reduction degree of the GA. Some research has investigated the synergistic effect of the hybrid nanofillers on PCMs [27,28,29,30]. Compared with a separate nanofiller, hybrid nanofillers endowed PCMs with enhanced performance. Zhang et al. [30] reported the preparation of a hybrid aerogel composed of graphene oxide and attapulgite aerogel. The fiber-bridged 3D network endowed stearic acid with outstanding heat storage performance. With an increase in the ATP content, the heat storage performance was enhanced. According to the research results, constructing a hybrid 3D structure is a promising route to achieve enhanced thermal performance of PCMs. However, it is still a challenge to achieve high thermal conductivity and high latent heat of PCMs.

In this paper, we successfully synthesized well-defined hybrid graphene aerogels (HGAs) with a 3D porous structure by hydrothermal reaction of GO and graphene nanoplatelets (GNPs). The HGA is expected to fully utilize the excellent thermal conductivity of GNPs (in-plane 3000 W/mK) and achieve a well-defined thermal conduction pathway. Herein, GO acted as a surfactant to disperse the GNPs in water due to the amphipathy of GO sheets. Thus, after the hydrothermal reaction, RGO nanosheets constructed well-developed thermal conductive pathways with GNPs dispersed uniformly along the network as thermal conductive fillers. Due to the synergistic effect of the GNPs and reduced GO network, the PA/HGA with GNPs exhibited enhanced thermal conductivity and latent heat compared with PA alone.

## 2. Materials and Methods

### 2.1. Materials

The palmitic acid was purchased from Sinopharm Chemical Reagent (Shanghai, China). Graphite oxide was purchased from the Sixth Element Ltd. (Changzhou, China). The GNPs were purchased from XG Sciences (Lansing, MI, USA). All the materials were used without further chemical treatment.

### 2.2. Preparation of HGA and PA/HGA

**Figure 1:**
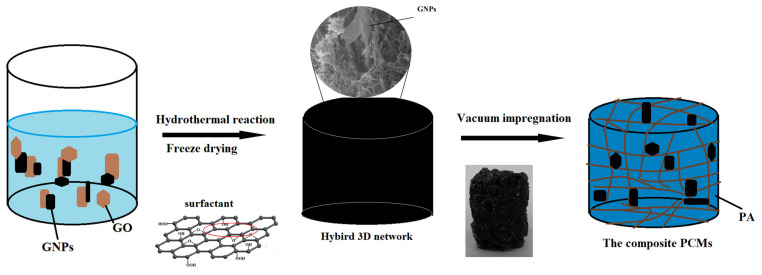
Schematic illustration of the preparation procedure of PA/HGA.

The preparation process of the PA/HGA is shown in Figure 1. Firstly, the graphite oxide was dispersed in deionized water. The aqueous suspension was sonicated for 2 h to obtain GO solution. Then, GO/GNPs solutions were individually placed in a Teflon-lined autoclave. The hydrothermal reaction condition was maintained at 180 °C for 24 h. During the hydrothermal reaction, GO was reduced by dehydration. Wrinkled RGO sheets overlapped and aggregated by π–π interactions with each other and formed a 3D supporting network structure. After the hydrothermal treatment, the obtained HGA samples with different GNPs content (0 wt%, 20 wt%, 30 wt%, 50 wt%) were freeze-dried and weighed and were marked as GA, HGA1, HGA2, and HGA3, respectively.

The PA/HGA was prepared via vacuum impregnation method. PA and HGA were heated to 80 °C in a vacuum oven. HGA was submerged in the melted PA. After the vacuum impregnation, the samples were removed and solidified at room temperature. The composited PCMs with different HGA contents (GA, HGA1, HGA2, and HGA3) were labeled PA/GA, PA/HGA1, PA/HGA2, and PA/HGA3, respectively. The samples were weighed. The content of the HGA and GNPs in the composite PCMs were calculated, and the results are shown in Table 1.

### 2.3. Characterization

A Magellan 400 FEI SEM (Hillsboro, OR, USA) was used to observe the morphology and microstructure of the HGA. X-ray photoelectron spectroscopy (XPS) was obtained by Perkin Elmer 5100c (Waltham, MA, USA) with Mg K radiation (h = 1253.6 eV). Atomic force microscopy (AFM) images were obtained using NSK SPA-300 HV (Tokyo, Japan). The X-ray diffractions (XRD) were measured using D/max2550VB3+/PC (Rigaku, Japan) with Cu Ka radiation. Fourier transform infrared (FTIR) spectrum was obtained using a BRUKER EQUINOXSS spectrometer (Karlsruhe, Germany). Thermogravimetric analysis (TGA) was performed using a Jupiter simultaneous thermal analyzer (STA 449 C, Selb, Germany) and was tested at a heating rate of 10 °C/min under nitrogen atmosphere. The modulated DSC curves were measured using a TA Instruments Q100 heat flux DSC (New Castle, DE, USA) at a heating rate of 3 °C/min [31,32]. All samples were tested under the same test condition. Reported results represent the average of three measurements for the same samples.

## 3. Results

### 3.1. Morphology and Structure of GO and GNPs

**Figure 2:**
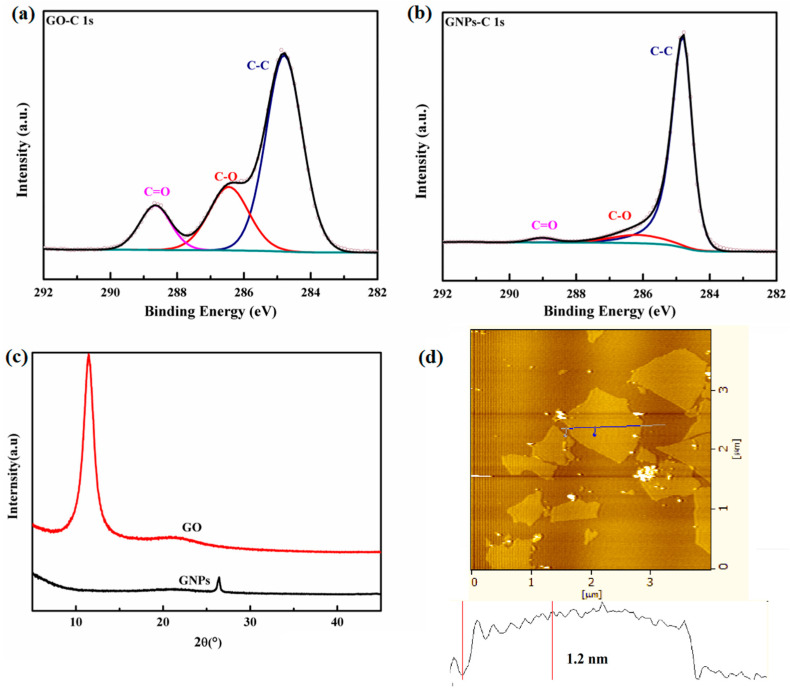
(**a**) XPS spectra of GO, (**b**) XPS spectra of GNPs, (**c**) FT-IR spectra of GO and GNPs, and (**d**) AFM image of GO with the height profile.

As illustrated in Figure 2a, in the C1s spectrum of GO, there are peaks in the C1s spectrum of GO. The C–C bond appears at 284.6 eV, and the peak at 286.5 eV is assigned to C–O and C=O appears at 288.5 eV. The C–O bond represents C–O–C and –OH. The C=O bond includes -C=O and –COOH. The XPS spectrum of GNPs (Figure 2b) exhibits weak intensities of these oxygen peaks. The C/O atomic ratio of GO and GNPs was 2.6 and 10.2, respectively. These indicates the existence of oxygen functional groups in GO. As shown in Figure 2c, the XRD pattern of GNPs shows a peak at 2θ = 26°, and the d-spacing is 3.36 Å. For GO, there is a sharp diffraction peak at 2θ about 11.2, while the characteristic peak at 2θ = 26° disappears. The d-spacing increased to 7.94 Å because of the presence of the oxygen-containing group. It shows that GO is successfully oxidized. The AFM image (Figure 2d) illustrates the thickness of GO sheet is about 1.2 nm, which is monolayer. The lateral size of GO is about 1~2 μm.

### 3.2. Morphology of HGA

**Figure 3:**
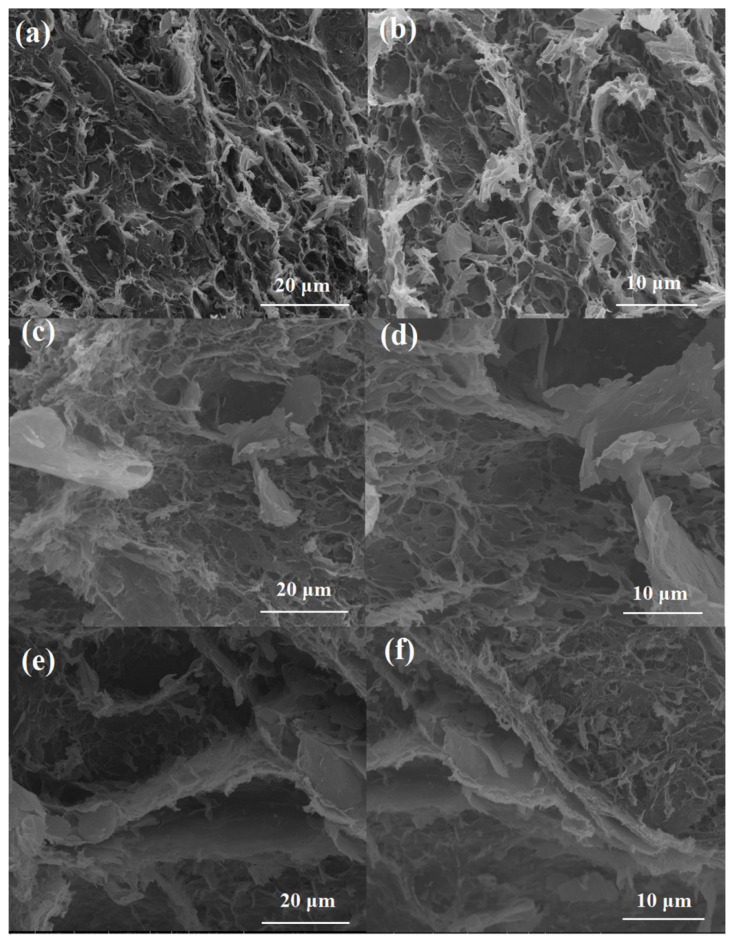
SEM images of GA (**a**,**b**), HGA1 (**c**,**d**), and HGA3 (**e**,**f**).

SEM images of the GA and HGA are shown in Figure 3. For GA without GNPs, wrinkled RGO sheets overlap with each other and form a 3D supporting network structure. The pore size is approximately several microns. The walls of the holes are a few layers of RGO sheets. The network provides a thermal conductive pathway. For HGA1 with 20 wt% GNPs, the GNPs present smooth surfaces with some creases, which are different from those of RGO (As shown in Figure 3b,d). It is found that GNPs are dispersed in the network structure of RGO. The GO sheets disperse GNPs in water as a surfactant because of the amphipathy of GO. The structure of GO has hydrophobic parts and π-conjugated parts. Thus, after the hydrothermal reaction, the RGO nanosheets form a 3D network, which provide a supporting structure. GNPs, as thermal conductive fillers, establish well-developed 3D thermal conductive pathways through the synergistic effect with GO. As shown in Figure 3e,f, with an increase in GNPs, the GNPs are more densely distributed in the 3D network skeleton. This is because the increase in GNP content leads to the poor dispersion of GNPs in the 3D network and the formation of the 3D GNPs’ network skeleton.

### 3.3. Chemical Structure of HGA and PA/HGA

**Figure 4:**
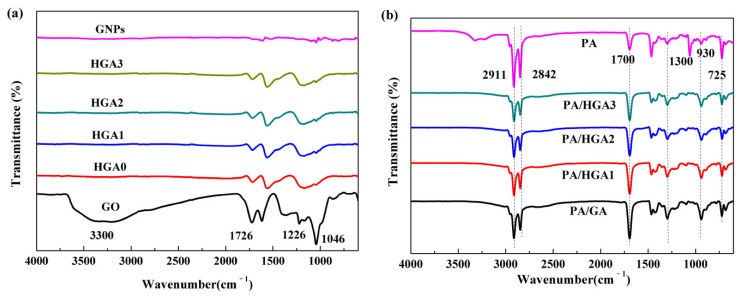
FT-IR spectrum of HGA (**a**) and PA/HGA (**b**).

Figure 4a illustrates the FT-IR spectra of GO, GNPs, and HGA. For GO, the peaks at 3330 cm^−1^ and 1396 cm^−1^ belong to the -OH stretching groups. The absorption peak at 1720 cm^−1^ is assigned to the C=O groups. C–O–C stretching vibrations are found at 1226 cm^−1^. The peak of the C–O group appears at 1045 cm^−1^. For GNPs, there are no strong peaks, which indicates that few oxygen functional groups exist in GNPs. After the hydrothermal reduction, for HGA, the intensities of the peaks corresponding to the –OH group disappear, and those corresponding to the other oxygen functional groups all decrease dramatically. No new absorption peaks appear. The results illustrate that the reduction degrees of the HGA after the hydrothermal reaction is similar. As presented in Figure 4b, the bands of PA at 2911 cm^−1^ and 2842 cm^−1^ are attributed to –CH_3_ and –CH_2_. The band at 1700 cm^−1^ indicates C=O stretching vibrations. The in-plane bending vibration of the –OH group appears at 1300 cm^−1^. The peaks at 930 cm^−1^ and 725 cm^−1^ represent the out-of-plane bending vibration and in-plane swinging vibration of the –OH functional group, respectively. No new peaks and no shifts of the peaks are observed in the PA/HGA, which suggests that only physical interactions occur between PA and HGA.

### 3.4. Thermal Properties of PA and PA/HGA

**Figure 5:**
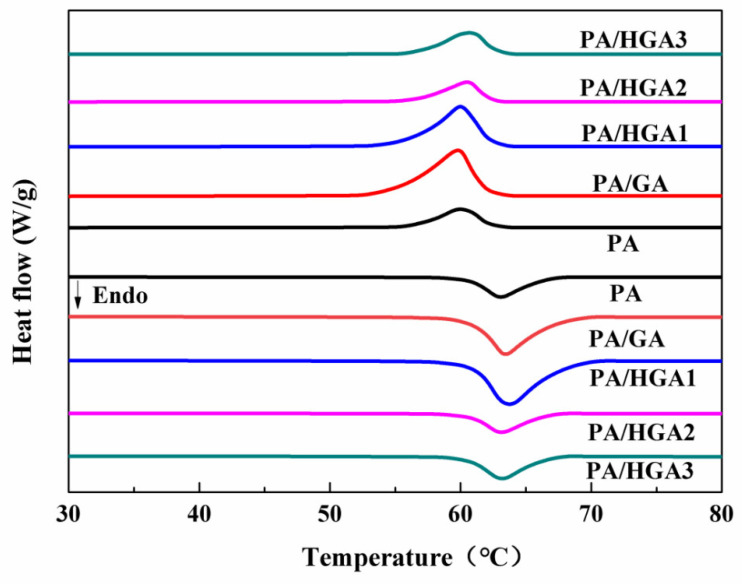
DSC scan of PA and PA/HGA.

**Figure 6:**
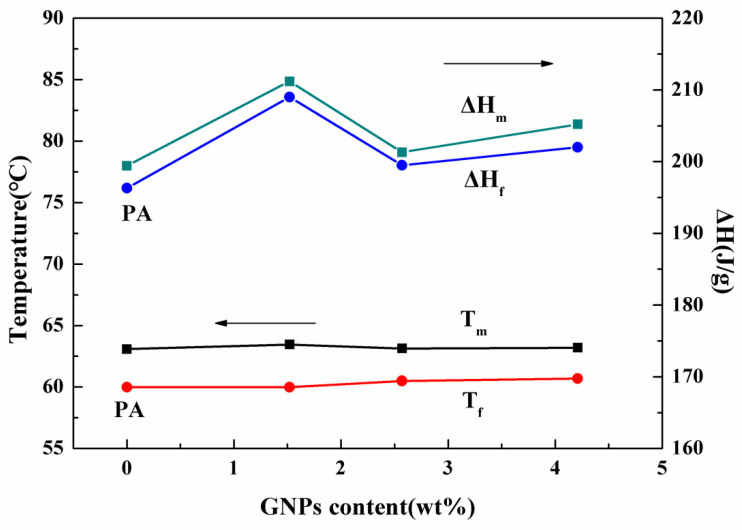
The phase-change temperature and latent heat of PA and PA/HGA with different GNPs content.

The phase-change temperature and latent heat are important to thermal performance. Figure 5 presents the DSC curves of PA and PA/HGA. The detailed DSC results (T_m_, ΔH_m_, T_f_, ΔH_f_) of the PA/HGA are depicted in Figure 6. There is one exothermic peak at 59.98 °C for the freezing process and one endothermic peak at 63.08 °C for the heating process of pure PA. For PA, the melting temperature (T_m_) and the freezing temperature (T_f_) of PA/GA are 63.59 °C and 60.45 °C, respectively. The T_m_ and T_f_ of the PA/HGA3 are 63.19 °C and 60.69 °C, respectively. Compared with those of PA, the T_m_ and the T_f_ increase slightly. These results show that the HGA promotes the crystallization process and restricts the solid-to-liquid transition of PA. The adsorption of the 3D graphene pore structure restricts the molecular movement of PA during the heating process. Meanwhile, HGA, as a nucleating agent, promotes the crystallization of the PA molecules during the cooling process.

The melting enthalpy (ΔH_m_) and the freezing enthalpy (ΔH_f_) of pure PA are 199.4 J/g and 196.3 J/g, respectively. The ΔH of the PA/HGA deviates little from PA, indicating that the presence of a graphene network, and GNPs in composite PCMs do not affect the thermal energy storage of PA. The PA/HGA3 also presents a high latent heat of 206.2 J/g. Although the ΔH of the PA/HGA increases, the change is not linear with the content of GNPs. Some studies have found that the ΔH was increased for the composite PCM [7,22,23,27]. According to the results, the high latent heat is related to the hybrid network structure and the intermolecular forces between HGA and PA. GNPs in the network act as a nucleating agent providing nucleation sites, which promotes the crystallization of PA molecules. The PA molecules confined by the hybrid network have a better degree of crystallization, while hybrid porous structures increase the interaction between HGA and PCMs by capillary and surface tension forces. The 3D hybrid porous structure restricts the molecular movement of PA, resulting in increased melting enthalpy.

### 3.5. Thermal Conductivity of PA and PA/HGA

**Figure 7:**
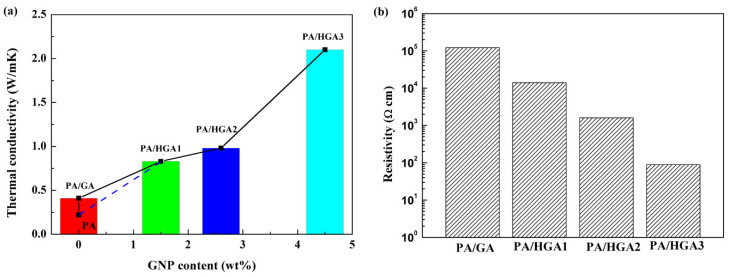
Thermal conductivity (**a**) and electrical resistivity (**b**) of PA and PA/HGA.

As exhibited in Figure 7a, PA shows low thermal conductivity (0.22 W/mK). The thermal conductivity of the PA/HGA is increased compared with that of PA. The thermal conductivity of PA/GA without GNPs is 0.41 W/mK, which shows 86.4% enhancement compared with that of PA. PA/HGA1 has a high conductivity of 0.83 W/mK, which is improved by 277% with a GNPs content of 1.5 wt%. These results indicate that the introduction of HGA is more effective on the thermal conductivity than GA because GNPs have a higher thermal conductivity than RGO reduced by the hydrothermal method. As a thermal conductive filler, GNPs plays an important role in improving the thermal conductivity of the composites. The thermal conductivity of PA/HGA2 and PA/HGA3 is 0.98 and 2.1 W/mK, which exhibits an increase of 345% and 854% over that of PA, respectively. The results reveal that the thermal conductivity of the PA/HGA increases with GNPs content. This is because that GNPs act as thermal conductive fillers. Three-dimensional thermal conductive pathways are established through the synergistic effect of GNPs and RGO. However, the thermal conductivity of PA/HGA2 shows a slower growing rate than that of PA/HGA1 because the high content of GNPs restricts the well dispersion of GNPs. The thermal conductivity is related to the GNPs content and the network structure. The poor dispersion of GNPs in the 3D network leads to the increase in thermal resistance. With the increase in GNP content, GNPs are more densely distributed and form the 3D GNP network skeleton (Figure 3e,f). Thus, the thermal conductivity keeps growing. However, we found it is hard to prepare a well dispersion of GO/GNPs with more GNPs. The well dispersion of high GNP content still needs to be further investigated.

As shown in Figure 7b, the electrical resistivity is significantly decreased from an electrical insulator to an electrical conductor. The resistivity of PA/GA is 10^5^ Ω cm. The electrical resistivity of the PA/HGA decreases when GNP content increases. The resistivity of PA/HGA3 is down to 89.5 Ω cm due to the presence of the conductive hybrid networks in the PCMs. The thermal conductivity and electrical resistivity of PA/HGA demonstrate the conductive pathway is constructed.

**Figure 8:**
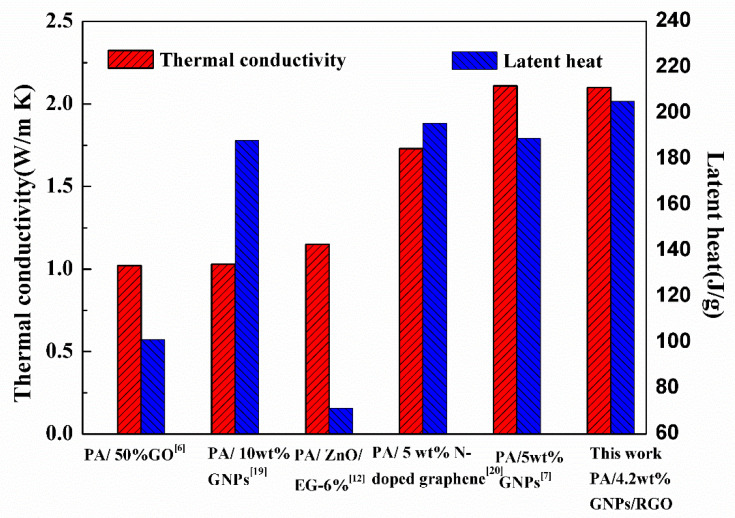
Thermal conductivity and latent heat of PA-based composites in the literature [6,7,12,19,20].

Thermal conductivity and latent heat are crucial parameters for PCMs. A comparison of the thermal performance parameter of the PA-based composites is demonstrated in Figure 8. The composite PCMs showed a remarkable increase in thermal conductivity (about 1~2 W/mK) at 4~10 wt% graphene. However, the latent heat value was decreased. This is because the content of PCMs as working materials is reduced. Graphene does not contribute to latent heat as it has no phase transition. This seems to be contradictory between thermal conductivity and heat latent values. For example, Mehrali et al. found PA with 5% GNPs had an enhanced thermal conductivity of (2.11 W/mK), which is improved by 627% compared with PA (0.29 W/mK) [7]. However, the latent heat of the PA/GNPs decreased to 188.98 kJ/kg from 205.53 kJ/kg (PA). In our work, PA/HGA3 shows high thermal conductivity (2.1 W/m K) and high latent heat, which are comparable to the previously reported performance. The thermally conductive pathway of HGA is constructed with the help of GO acting as a dispersing agent. Therefore, the thermal conductivity of the PCMs was improved remarkably. The high latent heat in this work is related to the hybrid network structure and the intermolecular forces between HGA and PCM. The DSC results show the phase-change behavior of PCMs is affected by the porous network structure. The T_m_ and the T_f_ of PA/HGA increase because GNPs, as a nucleating agent, increase the interaction between HGA and PCMs by capillary and surface tension forces. The forces restrict the solid-to-liquid transition, resulting in increased melting enthalpy. GNPs in the network act as a nucleating agent providing nucleation sites, which promotes the crystallization of PA molecules. The PA molecules confined by the hybrid network have a better degree of crystallization.

### 3.6. Thermal Stability of PA and PA/HGA

**Figure 9:**
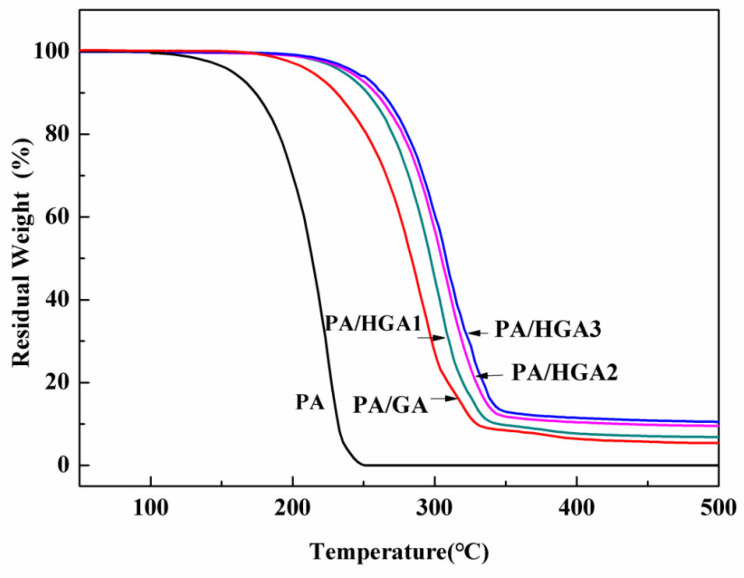
TGA curves of PA and PA/HGA.

Figure 9 exhibits the TGA results of PA and PA/HGA. The PA started to lose weight at 105 °C and is decomposed completely at 250 °C. For PA/HGA1, weight loss occurred at 165 °C, and the weight loss was rapid, from 165 °C to 350 °C, due to the thermal decomposition of the PA molecular chains. The weight loss temperature of the composite PCMs is delayed. The weight loss percentage is approximately 94.6%, 93.1%, 90.5%, and 89.5% for PA/GA, PA/HGA1, PA/HGA2, and PA/HGA3, respectively. The results demonstrate that the hybrid graphene network endows the PA with good thermal stability.

**Figure 10:**
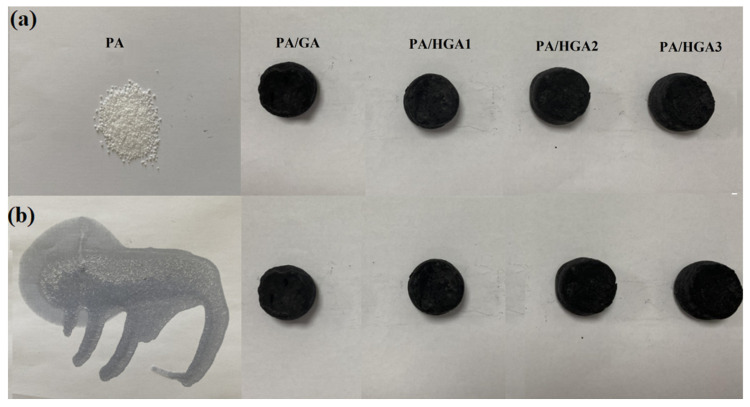
Photos of PA and PA/HGA heated at 80 °C for 0 min (**a**) and 30 min (**b**).

Figure 10 presents the photographs of PA and PA/HGA heated at 80 °C for 30 min. It can be seen that PA completely melts into liquid at 80 °C above its melting point. The composite PCMs show enhanced shape stability. Combined with the results in Figure 8, this may be attributed to the hybrid network structures of reduced GO and GNPs, which restrict the movement of the PA molecular chain.

**Figure 11:**
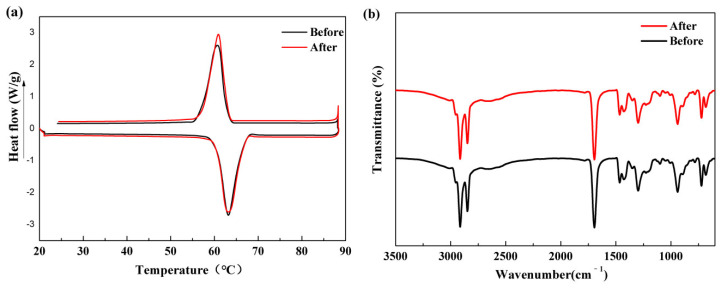
The DSC curves (**a**) and FT-IR spectrum (**b**) of PA/HGA3 before and after 100 thermal cycles.

Figure 11a exhibits the DSC curves of PA/HGA3 before and after 100 thermal cycles. The T_m_ and T_f_ values of PA/HGA3 change by −0.01 °C and 0.21 °C, respectively. The ΔH_m_ and ΔH_f_ values of PA/HGA3 are reduced by 1.2% and 2.3%. It can been seen from Figure 11b that the major peaks in the FT-IR spectra have no change. These results indicate that the composite PCMs have excellent thermal and chemical reliability.

## 4. Conclusions

In summary, HGAs have a 3D porous structure by the hydrothermal reaction of GO and GNPs. With the help of GO acting as a dispersing agent, GNPs dispersed along the RGO network as thermal conductive fillers. The HGA was introduced to PA, which brought PA/HGA with a new structure and enhanced thermal performance. With the increasing content of GNPs, the thermal conductivity of the PA/HGA composite significantly increased. The PA/HGA3 composite with 4.2 wt% GNPs exhibited enhanced thermal conductivity of 2.1 W/mK and an improvement of 831%. This composite also presents a high heat storage capacity of 206.2 J/g. The enhanced thermal conductivity is attributed to the GNPs content and the hybrid network structure, while the high latent heat is related to the hybrid network structure and the intermolecular forces between HGA and PCM. This research provides the possibility to optimize the thermal performance of the PCMs. To achieve high thermal conductivity and high latent heat, the well dispersion of high GNP content and more ideal HGA structure still need to be further investigated. Moreover, the effect of HGA on the thermal behaviors of PCMs should be systemically investigated by both experiment and theoretical investigations. Theoretical investigations of the interaction between graphene and PCMs will be helpful to the enhancement of comprehensive thermal performance. In addition, more comprehensive and in-depth research about the mechanism is needed.

## Figures and Tables

**Table 1:** The HGA, GNPs, and PA contents in PA/HGA.

Sample	HGA (wt%)	GNPs (wt%)	PA (wt%)
PA/GA	6.6(GA)	0	93.4
PA/HGA1	7.6	1.5	92.4
PA/HGA2	7.7	2.6	92.3
PA/HGA3	8.4	4.2	91.6

## Data Availability

Not applicable.
